# Supplementary material for: In situ electron paramagnetic resonance spectroscopy using single nanodiamond sensors
Source: Nat Commun. 2023 Oct 7;14:6278. doi: 10.1038/s41467-023-41903-5 (PMC10560202; doi:10.1038/s41467-023-41903-5)
Supplement: Supplementary file 1 — Supplementary Information [file 41467_2023_41903_MOESM1_ESM.pdf]

Supplementary Information for "In situ electron paramagnetic resonance spectroscopy using single nanodiamond sensors"

Supplementary Note 1. Brief description of the ND-EPR detection method

The Hamiltonian of the NV center with coupling to a target spin and the amplitude-modulated microwave can be written as

$$H = D(S_z^{\mathrm{NV}})^2 + \gamma_{\mathrm{NV}} B_1 \sin\theta \cos ft \cos Dt S_x^{\mathrm{NV}} + \sum_{i,j=\{x,y,z\}} d_{ij} S_i^{\mathrm{NV}} T_j + \omega T_z, \tag{1}$$

where $\mathbf{S}^{\mathrm{NV}}$ and $\mathbf{T}$ are the spin operator for the NV electron spin and the target spin, respectively, $D = 2.87$ GHz and $\gamma_{\mathrm{NV}} = -28.03$ GHz/T are the zero-field splitting and the gyromagnetic ratio of the NV electron spin, $\mathbf{B_1}$ is the magnetic field of the microwave, θ is the angle between $\mathbf{B_1}$ and the N-V axis, f is the amplitude-modulation frequency of the microwave, and d is the dipole-dipole coupling between the NV center and the target spin. In the interaction picture, the Hamiltonian becomes

$$\begin{aligned} H_{\mathrm{I}} &= e^{iDt(S_z^{\mathrm{NV}})^2} H e^{-iDt(S_z^{\mathrm{NV}})^2} - D(S_z^{\mathrm{NV}})^2 \\ &\approx \frac{\Omega}{2} \cos ft S_x^{\mathrm{NV}} + \sum_j d_{zj} S_z^{\mathrm{NV}} T_j + \omega T_z, \end{aligned} \tag{2}$$

where $\Omega = \gamma_{\mathrm{NV}} B_1 \sin\theta$ is the Rabi frequency, and we neglect the high-frequency items ($\Omega, f \ll D$). By moving to the second interaction picture and using the Jacobi-Anger expansion, the Hamiltonian becomes

$$\begin{aligned} H_{\mathrm{II}} &= e^{i\frac{\Omega}{2f}\sin ft S_x^{\mathrm{NV}}} H_{\mathrm{I}} e^{-i\frac{\Omega}{2f}\sin ft S_x^{\mathrm{NV}}} - \frac{\Omega}{2}\cos ft S_x^{\mathrm{NV}} \\ &= \sum_j d_{zj} [J_0(\frac{\kappa}{2}) S_z^{\mathrm{NV}} + \sum_{m=\mathrm{odd}} 2J_m(\frac{\kappa}{2}) \sin mft S_y^{\mathrm{NV}} + \sum_{n=\mathrm{even}} 2J_n(\frac{\kappa}{2}) \cos nft S_z^{\mathrm{NV}}] T_j + \omega T_z, \end{aligned} \tag{3}$$

where J_α is the α-th order Bessel functions of the first kind, and $\kappa = \Omega/f$ is the relative driving index. Here we assume the ND has a quasi-static tumbling, which means θ remain unchange during a single round of measurement. If $\Omega \ll f$, we can only keep up to the first order Bessel items, and then we have

$$H_{\mathrm{II}} \approx \sum_j d_{zj} \left(S_z^{\mathrm{NV}} + \frac{\kappa}{2} \sin ft S_y^{\mathrm{NV}} \right) T_j + \omega T_z. \tag{4}$$

We then move to the interaction picture of the target spin:

$$\begin{aligned} H_{\mathrm{III}} &= e^{iftT_z} H_{\mathrm{II}} e^{-iftT_z} - fT_z \\ &\approx d_{zz} S_z^{\mathrm{NV}} T_z + (\omega - f) T_z - \frac{\kappa}{4} d_{zx} S_y^{\mathrm{NV}} T_y + \frac{\kappa}{4} d_{zy} S_y^{\mathrm{NV}} T_x, \end{aligned} \tag{5}$$

where we again neglect the high-frequency items. Now we can see the resonance condition is $f = \omega \pm d_{zz}$, where the last item will induce a slight line splitting, which is negligible comparing to the line broadening. Therefore, the resonance condition can be well approximated as

$$f = \omega, \tag{6}$$

which dose not depend on Ω. At this condition, the Hamiltonian Supplementary Eq. 5 can be simplified to

$$H_{\mathrm{III}} = \frac{\kappa}{4} S_y^{\mathrm{NV}} (d_{zy} T_x - d_{zx} T_y), \tag{7}$$

where the effective sensor-target coupling strength reduce by a factor of $\kappa/4$. This coupling will induce a transition of the NV state from the initial $|0\rangle$, and thus a resonance spectrum can be detected by sweeping the amplitude-modulation frequency f. In our experiment, the sensor-target coupling is much weaker than the relaxation rate of the NV center $\Gamma_{2,\mathrm{NV}}$ and the target spin $\Gamma_{2,\mathrm{tar}}$, and thus the state transition of the NV center will degrade to an additional longitudinal relaxation, of which the rate is

$$\Gamma_1' = \frac{3\kappa^2 (d_{zx}^2 + d_{zy}^2)}{64} \frac{\Gamma_2}{{\Gamma_2}^2 + (f - \omega)^2}, \tag{8}$$

where $\Gamma_2 = \Gamma_{2,\mathrm{NV}} + \Gamma_{2,\mathrm{tar}}$ is the total relaxation rate. For an initial state $|0\rangle$ of the NV center, the probability of staying in $|0\rangle$ after a evolution time of t is

$$P_0(f,t) = \frac{1}{3} + \frac{2}{3}\exp[-(\Gamma_1 + \Gamma_1^{'})t], \tag{9}$$

where $\Gamma_1 = 1/T_{1,\mathrm{NV}}$ is the intrinsic longitudinal relaxation of the NV center. Therefore, the signal contrast is

$$S(f,t) = P_0(\infty,t) - P_0(f,t) = \frac{2}{3}e^{-\Gamma_1 t}(1 - e^{-\Gamma_1^{'} t}), \tag{10}$$

In the small signal limit, it can be simplified as

$$S(f,t) \approx \frac{\kappa^2(d_{zx}^2 + d_{zy}^2)}{32}\frac{\Gamma_2 t}{{\Gamma_2}^2 + (f-\omega)^2}e^{-\frac{t}{T_{1,\mathrm{NV}}}}, \tag{11}$$

Supplementary Note 2. Characterization of signal NDs

We keep reducing the density of the tethered NDs, until isolated spots can be resolved from the confocal microscope (Supplementary Fig. 1a). To further confirm the isolated spot is single NDs, we perform a scanning electron microscope (Supplementary Fig. 1b), where single NDs can be resolved. Supplementary Fig. 1c and D give the spin properties of the ND used in Fig. 3 in the main text.

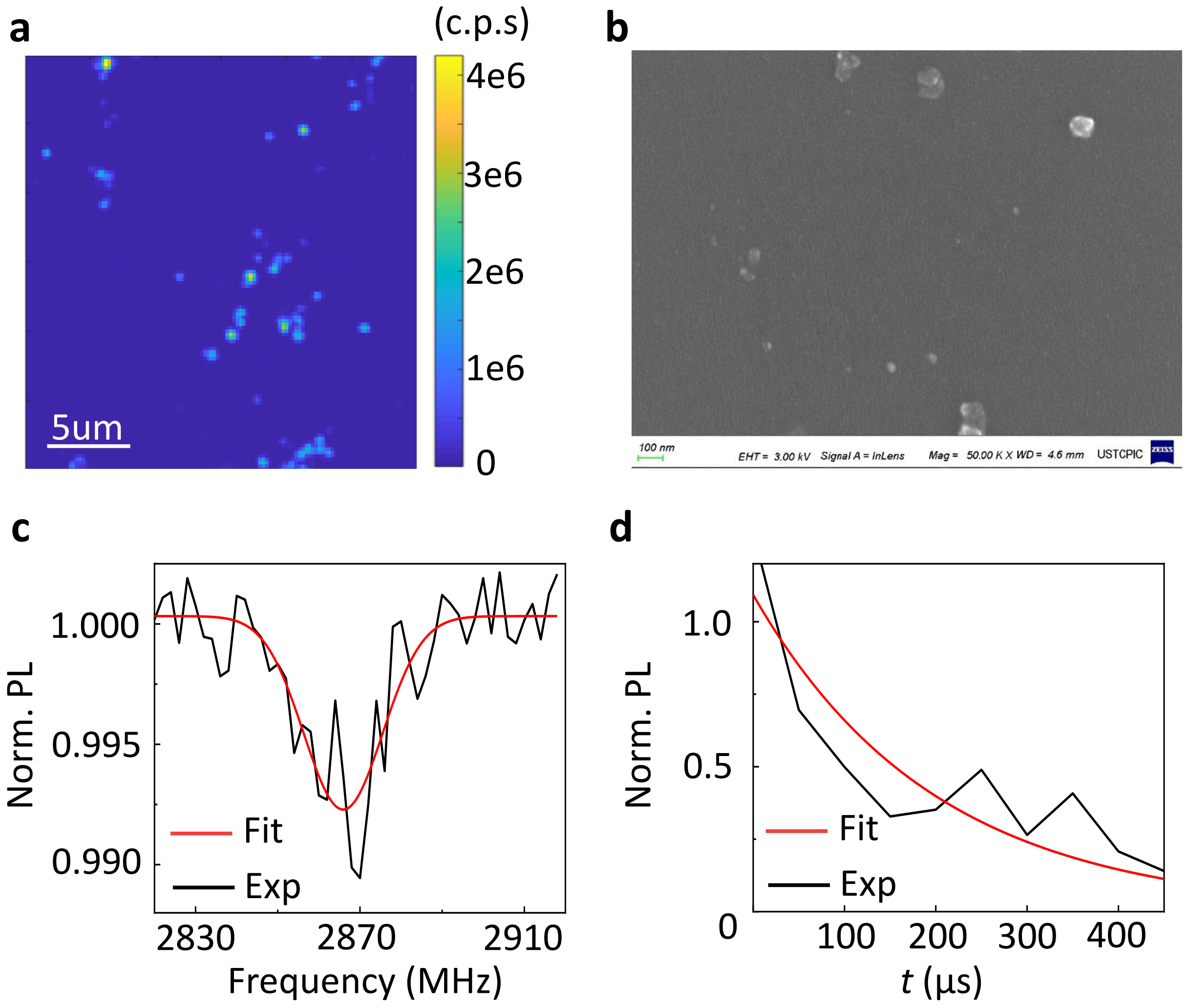

Supplementary Figure 1: **Characterization of the coverslip with tethered NDs.** **a** Confocal microscope. **b** Scanning electron microscope. **c** Optically detected magnetic resonance (ODMR) spectrum of the NV center itself. The fitted linewidth is 23 ± 2 MHz. **d** Longitudinal relaxation of the NV center. The fitted T_1 is 200 ± 40 μs.

Supplementary Note 3. ND-EPR signal of vanadyl ions

The spin Hamiltonian of the vanadyl ion (VO^{2+}) at zero magnetic field is

$$H_{\rm VO} = A_\perp(S_xI_x + S_yI_y) + A_\parallel S_zI_z + P[I_z^2 - \frac{1}{3}I(I+1)], \tag{12}$$

where $\mathbf{S}(S = 1/2)$ and $\mathbf{I}(I = 7/2)$ are the electron and nuclear spin operators, respectively, $A_\perp = 208.5$ MHz, $A_\parallel = 547$ MHz and $|P| = 0.2$ MHz are the hyperfine and nuclear quadrupole coupling constants. As P is too small, we neglect the quadrupole term for simplicity. The eigenstates can be directly written as $|T, m_T\rangle$ ($T = 4, 3, m_T = \pm T, \pm(T-1), \ldots, 0$), where $\mathbf{T} = \mathbf{S} + \mathbf{I}$ is the total angular momentum. In the $\{|m_S, m_I\rangle\}$ basis, we have

$$|T, m_T\rangle = \cos\frac{\alpha}{2}\left|\frac{1}{2}, m_T - \frac{1}{2}\right\rangle + \sin\frac{\alpha}{2}\left|-\frac{1}{2}, m_T + \frac{1}{2}\right\rangle, \tag{13}$$

where α depends on T, m_T, and $|\tan\alpha| = \chi_{m_T}/|m_T|$, as shown in Supplementary Fig. 2, where

$$\chi_{m_T} = \frac{A_\perp}{A_\parallel}\sqrt{I(I+1) - (m_T - \frac{1}{2})(m_T + \frac{1}{2})}.$$

The corresponding eigenenergies are

$$E_{T,m_T} = \begin{cases} \frac{A_\parallel}{4}(-1 + 2\sqrt{m_T^2 + \chi_{m_T}^2}), T = 4 \\ \frac{A_\parallel}{4}(-1 - 2\sqrt{m_T^2 + \chi_{m_T}^2}), T = 3 \end{cases}$$

Supplementary Fig. 2 gives the energy level structure, where all the $m_T \neq 0$ levels are doubly degenerate. For the specific case ($I = 7/2$) here, we have

$$\alpha(m_T) = \begin{cases} \arccos\frac{A_\parallel m_T}{\sqrt{A_\parallel^2 m_T^2 + A_\perp^2(16 - m_T^2)}}, & T = 4 \\ \pi + \arccos\frac{A_\parallel m_T}{\sqrt{A_\parallel^2 m_T^2 + A_\perp^2(16 - m_T^2)}}, & T = 3 \end{cases} \tag{14}$$

$$E_{T,m_T} = \begin{cases} -\frac{A_\parallel}{4} + \frac{1}{2}\sqrt{A_\parallel^2 m_T^2 + A_\perp^2(16 - m_T^2)}, T = 4 \\ -\frac{A_\parallel}{4} - \frac{1}{2}\sqrt{A_\parallel^2 m_T^2 + A_\perp^2(16 - m_T^2)}, T = 3 \end{cases} \tag{15}$$

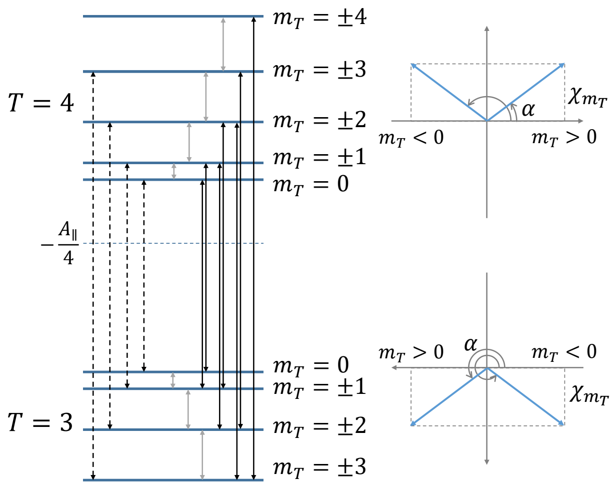

Supplementary Figure 2: **Diagram of the energy level structure of the vanadyl ion.**

A transition between $|T_0, m_{T_0}\rangle$ and $|T_1, m_{T_1}\rangle$ can happen by applying an ac magnetic field on either the electron spin or the nuclear spin with frequency of $\omega = E_{T_1,m_{T_1}} - E_{T_0,m_{T_0}}$. The field on nuclear is negligible because $\gamma_{\rm n}/\gamma_{\rm e} \sim 10^{-3}$,

so we focus on the electron spin. For a specific transition $|T_0, m_{T_0}\rangle \leftrightarrow |T_1, m_{T_1}\rangle$, the spin operator $\mathbf{S}$ can be reduced in the two-level subsystem as

$$\mathbf{S}^{\mathrm{r}} = \sum_{i,j=0,1} |T_i, m_{T_i}\rangle\langle T_i, m_{T_i}|(\mathbf{S}\otimes I)|T_j, m_{T_j}\rangle\langle T_j, m_{T_j}|. \tag{16}$$

After expansion, we have

$$\begin{aligned} S^{\mathrm{r}}_x &= \left[\sin\frac{\alpha_0}{2}\cos\frac{\alpha_1}{2}\delta(m_{T_1}-m_{T_0}-1)+\cos\frac{\alpha_0}{2}\sin\frac{\alpha_1}{2}\delta(m_{T_1}-m_{T_0}+1)\right]T_x,\\ S^{\mathrm{r}}_y &= \left[\sin\frac{\alpha_0}{2}\cos\frac{\alpha_1}{2}\delta(m_{T_1}-m_{T_0}-1)-\cos\frac{\alpha_0}{2}\sin\frac{\alpha_1}{2}\delta(m_{T_1}-m_{T_0}+1)\right]T_y,\\ S^{\mathrm{r}}_z &= \cos\frac{\alpha_0+\alpha_1}{2}\delta(m_{T_1}-m_{T_0})T_x+\frac{1}{2}(\cos\alpha_1-\cos\alpha_0)T_z. \end{aligned} \tag{17}$$

Therefore, the selection rule of transition is $\Delta m_T = 0, \pm 1$. The reduction can be written in a simple form

$$\mathbf{S}^T = \mathcal{M}_{\Delta m_T}\cdot\mathbf{T}^T, \tag{18}$$

where

$$\mathcal{M}_0 = \cos\frac{\alpha_0+\alpha_1}{2}\begin{pmatrix}0&0&0\\0&0&0\\1&0&*\end{pmatrix}, \mathcal{M}_1 = \sin\frac{\alpha_0}{2}\cos\frac{\alpha_1}{2}\begin{pmatrix}1&0&0\\0&1&0\\0&0&*\end{pmatrix}, \mathcal{M}_{-1} = \cos\frac{\alpha_0}{2}\sin\frac{\alpha_1}{2}\begin{pmatrix}1&0&0\\0&-1&0\\0&0&*\end{pmatrix},$$

and $*$ corresponds to negligible T_z-related couplings.

For the sensor-target system, the ac field is coming from the dipole-dipole coupling between the NV center and the vanadyl ion, which is

$$H_{\mathrm{dd}} = \frac{\mu_0\gamma_{\mathrm{NV}}\gamma_{\mathrm{e}}\hbar}{4\pi r^3}\left[\mathbf{S}^{\mathrm{NV}}\cdot\mathcal{R}\cdot\mathbf{S}^T - 3(\mathbf{S}^{\mathrm{NV}}\cdot\hat{\mathbf{r}}^T)(\hat{\mathbf{r}}\cdot\mathcal{R}\cdot\mathbf{S}^T)\right], \tag{19}$$

where γ_{e} is the gyromagnetic ratios of the vanadyl electron spin, $\mathbf{r} = r(\sin\theta_r\cos\phi_r, \sin\theta_r\sin\phi_r, \cos\theta_r)$ is the separation vector between the NV center and the vanadyl ion, and

$$\mathcal{R} = \begin{pmatrix}\cos\theta_{\mathrm{e}}\cos\phi_{\mathrm{e}} & -\sin\phi_{\mathrm{e}} & \sin\theta_{\mathrm{e}}\cos\phi_{\mathrm{e}}\\ \cos\theta_{\mathrm{e}}\sin\phi_{\mathrm{e}} & \cos\phi_{\mathrm{e}} & \sin\theta_{\mathrm{e}}\sin\phi_{\mathrm{e}}\\ -\sin\theta_{\mathrm{e}} & 0 & \cos\theta_{\mathrm{e}}\end{pmatrix}$$

is the transformation matrix from the principle-axis frame of the vanadyl ion to the NV frame. Therefore, the dipole-dipole coupling matrix becomes

$$\mathcal{D} = \frac{\mu_0\gamma_{\mathrm{NV}}\gamma_{\mathrm{e}}\hbar}{4\pi r^3}(I_3 - 3\hat{\mathbf{r}}^T\cdot\hat{\mathbf{r}})\cdot\mathcal{R}\cdot\mathcal{M}, \tag{20}$$

where I_3 is a 3-dimensional identity matrix. As given in Supplementary Eq. 7, only $d_{zx} = \mathcal{D}_{31}$ and $d_{zy} = \mathcal{D}_{32}$ contribute to the signal.

Calculation of the absolute signal strength depends on the specific position and orientation of the target spin, so we first focus on the relative signal strength related to the reduction matrix $\mathcal{M}$:

$$\xi = \begin{cases}\cos^2\frac{\alpha_0+\alpha_1}{2}, & \Delta m_T = 0\\ \sin^2\frac{\alpha_0}{2}\cos^2\frac{\alpha_1}{2}, & \Delta m_T = 1\\ \cos^2\frac{\alpha_0}{2}\sin^2\frac{\alpha_1}{2}, & \Delta m_T = -1\end{cases} \tag{21}$$

The transitions of vanadyl ions can be divided into 3 categories, corresponding to 3 different kinds of arrows in Supplementary Fig. 2:

1. $\Delta T = 1$, $\Delta m_T = 0$. There are 7 transitions, of which the transition frequencies are

$$\omega(m_T) = \sqrt{A_\parallel^2 m_T^2 + A_\perp^2(16-m_T^2)}, m_T = 0, \pm1, \pm2, \pm3. \tag{22}$$

The corresponding relative signal strength is

$$\begin{aligned}\xi(m_T) &= \cos^2\frac{\alpha_0+\alpha_1}{2} = \cos^2\frac{\alpha_0+\alpha_0+\pi}{2} = \sin^2\alpha_0 \\ &= \frac{A_\perp^2(16-m_T^2)}{A_\parallel^2 m_T^2 + A_\perp^2(16-m_T^2)}\end{aligned} \tag{23}$$

Note that $\omega(-m_T)=\omega(m_T)$, so only 4 resonance peaks are observable. The specific frequencies and strength are

No.	frequency ω	strength ξ
1	$4A_\perp$	1
2	$\sqrt{A_\parallel^2+15A_\perp^2}$	$\frac{30A_\perp^2}{A_\parallel^2+15A_\perp^2}$
3	$\sqrt{4A_\parallel^2+12A_\perp^2}$	$\frac{24A_\perp^2}{4A_\parallel^2+12A_\perp^2}$
4	$\sqrt{9A_\parallel^2+7A_\perp^2}$	$\frac{14A_\perp^2}{9A_\parallel^2+7A_\perp^2}$

2. $\Delta T=0$, $\Delta m_T=\pm1$. There are 14 transitions. Since $|T,m_T\rangle \leftrightarrow |T,m_T+1\rangle$ and $|T,-m_T\rangle \leftrightarrow |T,-m_T-1\rangle$ have the same frequency, only 4 resonance peaks are observable. The transition frequencies are

$$\omega(m_T)=\frac{1}{2}\left[\sqrt{A_\parallel^2(m_T+1)^2+A_\perp^2(16-(m_T+1)^2)}-\sqrt{A_\parallel^2 m_T^2+A_\perp^2(16-m_T^2)}\right], m_T=0,1,2,3. \tag{24}$$

For $m_T=0,1,2$, each frequency corresponding to 4 transitions, and the sum relative signal strength is

$$\xi(m_T)=1-\cos\alpha(m_T)\cos\alpha(m_T+1), m_T=0,1,2. \tag{25}$$

For $m_T=3$, there are only 2 transitions, and the sum relative signal strength is

$$\xi(m_T=3)=(1-\cos\alpha(m_T))(1+\cos\alpha(m_T+1))/2. \tag{26}$$

The specific frequencies and strength are

No.	frequency ω	strength ξ
5	$\frac{1}{2}\left[\sqrt{A_\parallel^2+15A_\perp^2}-4A_\perp\right]$	1
6	$\frac{1}{2}\left[\sqrt{4A_\parallel^2+12A_\perp^2}-\sqrt{A_\parallel^2+15A_\perp^2}\right]$	$1-\frac{2A_\parallel^2}{\sqrt{A_\parallel^2+15A_\perp^2}\sqrt{4A_\parallel^2+12A_\perp^2}}$
7	$\frac{1}{2}\left[\sqrt{9A_\parallel^2+7A_\perp^2}-\sqrt{4A_\parallel^2+12A_\perp^2}\right]$	$1-\frac{6A_\parallel^2}{\sqrt{4A_\parallel^2+12A_\perp^2}\sqrt{9A_\parallel^2+7A_\perp^2}}$
8	$\frac{1}{2}\left[4A_\parallel-\sqrt{9A_\parallel^2+7A_\perp^2}\right]$	$1-\frac{3A_\parallel}{\sqrt{9A_\parallel^2+7A_\perp^2}}$

3. $\Delta T=1$, $\Delta m_T=\pm1$. There are 14 transitions. Similarly, only 4 resonance peaks are observable. The transition frequencies are

$$\omega(m_T)=\frac{1}{2}\left[\sqrt{A_\parallel^2(m_T+1)^2+A_\perp^2(16-(m_T+1)^2)}+\sqrt{A_\parallel^2 m_T^2+A_\perp^2(16-m_T^2)}\right], m_T=0,1,2,3. \tag{27}$$

The corresponding relative signal strength is

$$\xi(m_T)=\begin{cases}1+\cos\alpha(m_T)\cos\alpha(m_T+1), & m_T=0,1,2\\ (1+\cos\alpha(m_T))(1+\cos\alpha(m_T+1))/2, & m_T=3\end{cases} \tag{28}$$

The specific frequencies and strength are

No.	frequency ω	strength ξ
9	$\frac{1}{2}\left[\sqrt{A_\parallel^2+15A_\perp^2}+4A_\perp\right]$	1
10	$\frac{1}{2}\left[\sqrt{4A_\parallel^2+12A_\perp^2}+\sqrt{A_\parallel^2+15A_\perp^2}\right]$	$1+\frac{2A_\parallel^2}{\sqrt{A_\parallel^2+15A_\perp^2}\sqrt{4A_\parallel^2+12A_\perp^2}}$
11	$\frac{1}{2}\left[\sqrt{9A_\parallel^2+7A_\perp^2}+\sqrt{4A_\parallel^2+12A_\perp^2}\right]$	$1+\frac{6A_\parallel^2}{\sqrt{4A_\parallel^2+12A_\perp^2}\sqrt{9A_\parallel^2+7A_\perp^2}}$
12	$\frac{1}{2}\left[4A_\parallel+\sqrt{9A_\parallel^2+7A_\perp^2}\right]$	$1+\frac{3A_\parallel}{\sqrt{9A_\parallel^2+7A_\perp^2}}$

For simplicity, we assume that the ND is spherical with radius of a_0. The origin of the coordinates locates at the center of the sphere with the z axis along the N-V axis and the xz plane containing the NV center. As shown in Supplementary Fig. 3a, the NV center locates at $\mathbf{r}_{\rm NV} = r_{\rm NV}(\sin\theta, 0, \cos\theta)$, while the vanadyl ion locates at $\mathbf{r}_{\rm VO} = r_{\rm VO}(\sin\theta_r\cos\phi_r, \sin\theta_r\sin\phi_r, \cos\theta_r)$. So the separation vector between the NV center and the vanadyl ion is

$$\mathbf{r} = \mathbf{r}_{\rm VO} - \mathbf{r}_{\rm NV} = (r_{\rm VO}\sin\theta_r\cos\phi_r - r_{\rm NV}\sin\theta, r_{\rm VO}\sin\theta_r\sin\phi_r, r_{\rm VO}\cos\theta_r - r_{\rm NV}\cos\theta). \tag{29}$$

Substituting Supplementary Eq. 29 into Supplementary Eq. 20 yields the final dipole-dipole coupling matrix for calculating the EPR signal.

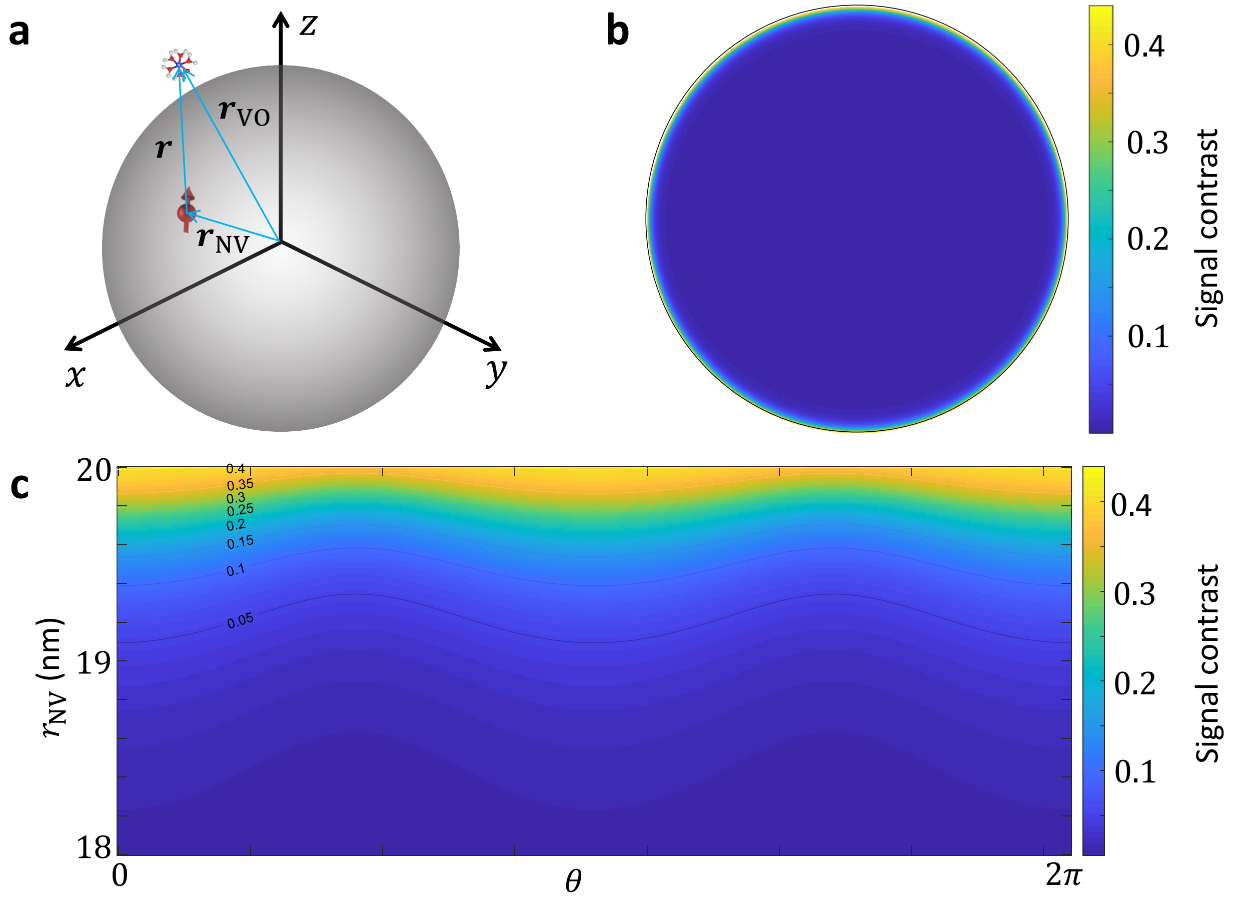

Supplementary Figure 3: **Calculations of the ND-EPR signal.** **a** Model of the sensor-target system. **b** Dependence of the EPR signal of transition 10 ($|3,\pm1\rangle \leftrightarrow |4,\pm2\rangle$, $|3,\pm2\rangle \leftrightarrow |4,\pm1\rangle$) on the position of NV center. The parameters for calculations are: $a_0 = 20$ nm, $r_0 = 0.37$ nm, $\kappa = 0.15$, $T_{1,\rm NV} = 200$ μs, $t = 100$ μs, $\Gamma_2 = 20$ MHz, and $c = 25$ mM. **c** Zoom in of the signal distribution in polar coordinate system.

In our experiment, the vanadyl ion has random transitional and rotational motions. So the resonant $\Gamma_1^{'}$ is averaged over all positions and orientations of the vanadyl ion:

$$\begin{aligned}\langle\Gamma_1^{'}\rangle &= \int_a^\infty\int_0^\pi\int_0^{2\pi}\int_0^\pi\int_0^{2\pi}\frac{1}{8}\frac{3\kappa^2(\mathcal{D}_{31}^2+\mathcal{D}_{32}^2)}{64\Gamma_2}\cdot c\cdot 4\pi r_{\rm VO}^2 dr_{\rm VO}\frac{\sin\theta_{\rm e}}{2}d\theta_{\rm e}\frac{1}{2\pi}d\phi_{\rm e}\frac{\sin\theta_r}{2}d\theta_r\frac{1}{2\pi}d\phi_r \\ &= \left[\frac{(1+\cos^2\theta)a^3}{(a^2-r_{\rm NV}^2)^3}+\frac{(1-3\cos^2\theta)a(a^2+r_{\rm NV}^2)}{8r_{\rm NV}^2(a^2-r_{\rm NV}^2)^2}+\frac{(3\cos^2\theta-1)arctanh\frac{r_{\rm NV}}{a}}{8r_{\rm NV}^3}\right]\frac{\beta\xi c(\mu_0\gamma_{\rm NV}\gamma_{\rm e}\hbar\kappa)^2}{4096\pi\Gamma_2},\end{aligned} \tag{30}$$

where the extra coefficient of 1/8 is the probability of a specific transition $|T_0, m_{T_0}\rangle \leftrightarrow |T_1, m_{T_1}\rangle$ happening, $a = a_0 + r_0$ is the minimum $r_{\rm VO}$ considering the radius of the vanadyl ion r_0, c is the concentration of the vanadyl ion, and $\beta = 1, 2$ for $\Delta m_T = 0, \pm1$, respectively. To clearly see the dependence on $r_{\rm NV}$ or the NV depth $h = a - r_{\rm NV}$, we focus on the

signal averaged over θ:

$$\langle S\rangle = \frac{2t}{3}e^{-\Gamma_1 t}\int_0^{\pi}\langle\Gamma_1^{'}\rangle\frac{\sin\theta}{2}d\theta = \frac{\beta\xi c t a^3(\mu_0\gamma_{\mathrm{NV}}\gamma_{\mathrm{e}}\hbar\kappa)^2}{4608\pi\Gamma_2(a+r_{\mathrm{NV}})^3h^3}e^{-\Gamma_1 t} \approx \frac{\beta\xi c t(\mu_0\gamma_{\mathrm{NV}}\gamma_{\mathrm{e}}\hbar\kappa)^2}{4608\pi\Gamma_2 h^3}e^{-\Gamma_1 t}, \tag{31}$$

where the approximation is made when $h \ll a$. Therefore, for shallow NV centers, the EPR signal has $\sim h^{-3}$ dependence on the NV depth h.

We take transition 10 ($|3,\pm1\rangle \leftrightarrow |4,\pm2\rangle$, $|3,\pm2\rangle \leftrightarrow |4,\pm1\rangle$) as an example, and calculate the dependence of the EPR signal on the position of the NV center. As shown in Supplementary Fig. 3b, the signal decreases dramatically with increasing NV depth. For the experimental result in Fig. 3b in the main text, the signal of transition 10 is roughly 0.05, which is normalized by the Rabi contrast of 0.1. It corresponds to a NV depth of $\sim$ 1 nm (Supplementary Fig. 3c). Such a shallow NV center should have unstable charge states. Therefore, this simple model seems insufficient to explain the experimental result. If there exists an adsorption layer on the ND surface with local ion concentration higher than that in the solution, deeper NV centers can explain the detected signal.

Supplementary Note 4. Alternative scheme for ND-EPR

The amplitude-modulated microwave consists of two detuned parts:

$$B_1 \cos ft \cos Dt = \frac{B_1}{2}\cos(D+f)t + \frac{B_1}{2}\cos(D-f)t.$$

Either of them can be alone used for EPR detection. Without loss of generality, we take the low-frequency part as an example. The Hamiltonian of the sensor-target system is

$$H = D(S_z^{\mathrm{NV}})^2 + \gamma_{\mathrm{NV}} B_1 \sin\theta \cos(D-f)t S_x^{\mathrm{NV}} + \sum_{i,j=\{x,y,z\}} d_{ij} S_i^{\mathrm{NV}} T_j + \omega T_z. \tag{32}$$

In the interaction picture, the Hamiltonian becomes

$$\begin{aligned} H_{\mathrm{I}} &= e^{i(D-f)t(S_z^{\mathrm{NV}})^2} H e^{-i(D-f)t(S_z^{\mathrm{NV}})^2} - (D-f)(S_z^{\mathrm{NV}})^2 \\ &\approx f S_z^{\mathrm{NV}} + \frac{\Omega}{2} S_x^{\mathrm{NV}} + \sum_j d_{zj} S_z^{\mathrm{NV}} T_j + \omega T_z. \end{aligned} \tag{33}$$

To better see the sensing process, we diagonalize the NV Hamiltonian as

$$\begin{aligned} H_{\mathrm{I}}^R &= R H_{\mathrm{I}} R^{-1} \\ &= \sqrt{f^2+\Omega^2}\begin{pmatrix} \cos\varphi & & \\ & -\sin^2\frac{\varphi}{2} & \\ & & \cos^2\frac{\varphi}{2} \end{pmatrix} + \sum_j d_{zj} \begin{pmatrix} 0 & \sin\frac{\varphi}{2} & \cos\frac{\varphi}{2} \\ \sin\frac{\varphi}{2} & 0 & 0 \\ \cos\frac{\varphi}{2} & 0 & 0 \end{pmatrix} T_j + \omega T_z, \end{aligned} \tag{34}$$

where

$$R = \begin{pmatrix} \frac{1}{\sqrt{2}} & 0 & -\frac{1}{\sqrt{2}} \\ \frac{1}{\sqrt{2}}\sin\frac{\varphi}{2} & -\cos\frac{\varphi}{2} & \frac{1}{\sqrt{2}}\sin\frac{\varphi}{2} \\ \frac{1}{\sqrt{2}}\cos\frac{\varphi}{2} & \sin\frac{\varphi}{2} & \frac{1}{\sqrt{2}}\cos\frac{\varphi}{2} \end{pmatrix}, \varphi = \arctan\kappa, \text{and } \kappa = \frac{\Omega}{f}.$$

In the case of $\kappa \ll 1$ and weak coupling, the Hamiltonian can be simplified as

$$H_{\mathrm{I}}^R \approx \begin{pmatrix} f & & \\ & -\frac{1}{4}\kappa^2 f & \\ & & f+\frac{1}{4}\kappa^2 f \end{pmatrix} + \frac{\kappa}{2}\begin{pmatrix} 0 & 1 & 0 \\ 1 & 0 & 0 \\ 0 & 0 & 0 \end{pmatrix}(d_{zx}T_x + d_{zy}T_y) + \omega T_z, \tag{35}$$

A resonant flip-flop process will happen at

$$f = (1 - \frac{\kappa^2}{4})\omega. \tag{36}$$

Different from the amplitude-modulated scheme, the resonance condition now has second-order dependence on Ω (Supplementary Fig. 4a). The resonance peak will have a slight redshift, and thus line broadening if the ND is tumbling (Supplementary Fig. 4b). These drawbacks are obvious when the spectrum has a narrow linewidth, such as measuring some paramagnetic species with long coherence time using high-profile NV centers. On the other hand, these drawbacks are negligible when the spectrum itself has a large linewidth (Supplementary Fig. 4cd). For example, the maximum shift for the spectrum of vanadyl ions in the main text is ~ 6 MHz ($\kappa < 0.15$), which is much smaller than the linewidth ~ 50 MHz. So the off-resonance scheme has no obvious difference from the amplitude-modulated scheme in this case. Similarly, the signal contrast will be

$$S(f,t) \approx \frac{\kappa^2(d_{zx}^2 + d_{zy}^2)}{16}\frac{\Gamma_2 t}{{\Gamma_2}^2 + (f-\omega)^2} e^{-\frac{t}{T_{1,\mathrm{NV}}}} \tag{37}$$

Considering the imperfection of the microwave devices, the off-resonance scheme is sometimes technically simpler. For example, the driving microwave has strong high-order harmonics (Supplementary Fig. 5a). If considering one

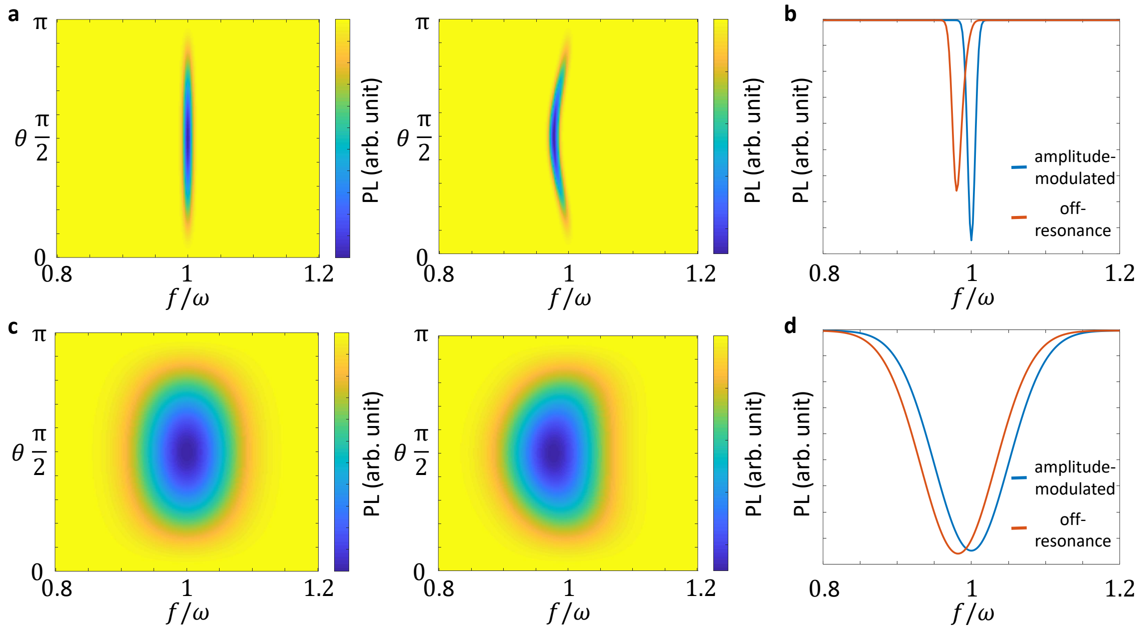

Supplementary Figure 4: **Comparison between the amplitude-modulated scheme and the off-resonance scheme. a** Simulated EPR spectra for amplitude-modulated (left) and off-resonance (right) schemes and the dependence on ND orientations. **b** Expected spectra after average over random orientations. **c**,**d** Similar with **a**,**b** but with 10 times larger linewidth.

detuned part of the amplitude-modulated microwave as the off-resonant driving field and the other part as the artificial signal field, a resonance will happen at

$$|n_1(D+f) \pm n_2(D-f)| = D, \tag{38}$$

where $n_{1,2} = 1, 2, 3, \cdots$. Therefore, artifact peaks will appear at 718 MHz ($n_1 = 2, n_2 = 2$), 1148 MHz ($n_1 = 2, n_2 = 3$), and so on (Supplementary Fig. 5b). Fortunately, these peaks are always much narrower than the spectrum of vanadyl ions. For example, the artifact peak at 1148 MHz is $2D/5$, corresponding to a linewidth of $4\Gamma_2/5 \sim 8$ MHz. So these artifact peaks can be carefully avoided. A special case is the peak at 1435 MHz ($n_1 = 0, n_2 = 2$), which is the artifact of the driving field itself.

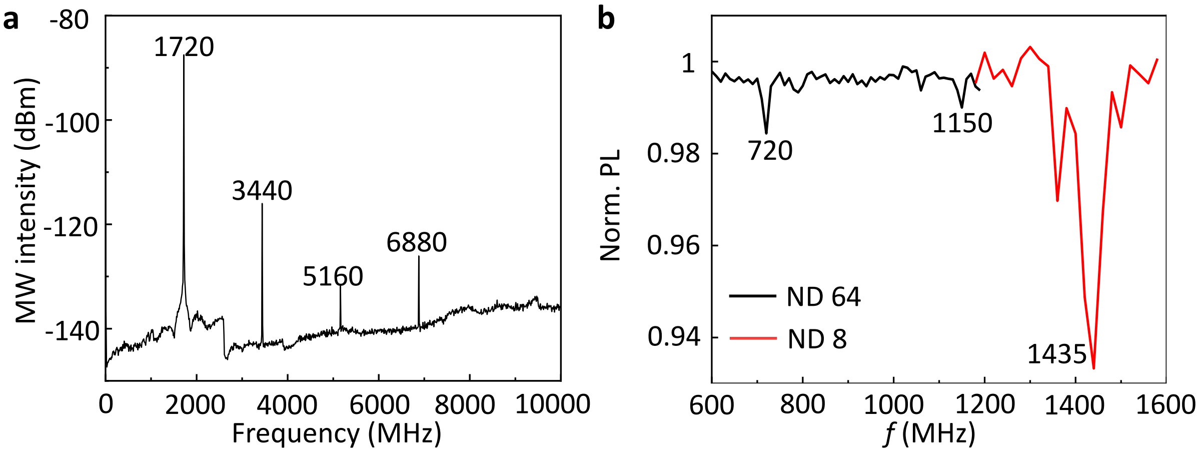

Supplementary Figure 5: **Artifacts induced by high-order harmonics. a** Frequency spectrum of a output microwave with a 1720 MHz input microwave. The data is measured by a microwave analyzer (Keysight N9917A). **b** Zero-field EPR measurement performed on a blank ND. The strong peaks appeared at 720, 1150, and 1435 MHz are artifacts induced by the driving field.

Supplementary Note 5. Effects of axial microwave

If consider the axial microwave, the Hamiltonian of the sensor-target system is

$$H = D(S_z^{\rm NV})^2 + \gamma_{\rm NV} B_1 \cos{(D-f)t} (\sin\theta S_x^{\rm NV} + \cos\theta S_z^{\rm NV}) + \sum_{i,j=\{x,y,z\}} d_{ij} S_i^{\rm NV} T_j + \omega T_z. \tag{39}$$

In interaction picture, the Hamiltonian becomes

$$\begin{aligned} H_{\rm I} &= e^{i\left[(D-f)t(S_z^{\rm NV})^2 + \frac{\Omega_z}{D-f}\sin ft S_z^{\rm NV}\right]} H e^{-i\left[(D-f)t(S_z^{\rm NV})^2 + \frac{\Omega_z}{D-f}\sin ft S_z^{\rm NV}\right]} - (D-f)(S_z^{\rm NV})^2 - \Omega_z \cos(D-f)t S_z^{\rm NV} \\ &\approx f S_z^{\rm NV} + \frac{\Omega_x}{2} J_0(\frac{\Omega_z}{D-f}) S_x^{\rm NV} + \sum_j \left[d_{xj} J_1(\frac{\Omega_z}{D-f}) S_{xx}^{\rm NV} + d_{yj} J_1(\frac{\Omega_z}{D-f}) S_{yy}^{\rm NV} + d_{zj} S_z^{\rm NV} \right] T_j + \omega T_z, \end{aligned} \tag{40}$$

where $\Omega_x = \gamma_{\rm NV} B_1 \sin\theta$, $\Omega_z = \gamma_{\rm NV} B_1 \cos\theta$, and

$$S_{xx}^{\rm NV} = \begin{pmatrix} 0 & -1 & 0 \\ -1 & 0 & 1 \\ 0 & 1 & 0 \end{pmatrix}, S_{yy}^{\rm NV} = \begin{pmatrix} 0 & i & 0 \\ -i & 0 & -i \\ 0 & i & 0 \end{pmatrix}.$$

Similar with Sec. S3, the Hamiltonian can be simplified as

$$H_{\rm I}^R \approx \begin{pmatrix} f & & \\ & -\frac{\Omega_x^2}{4f} & \\ & & f + \frac{\Omega_x^2}{4f} \end{pmatrix} + \sum_j \begin{pmatrix} 0 & \frac{\Omega_x d_{zj}}{2f} + \frac{\Omega_z d_{xj}}{2(D-f)} & 0 \\ \frac{\Omega_x d_{zj}}{2f} + \frac{\Omega_z d_{xj}}{2(D-f)} & 0 & i \cdot \frac{\Omega_z d_{yj}}{2(D-f)} \\ 0 & -i \cdot \frac{\Omega_z d_{yj}}{2(D-f)} & 0 \end{pmatrix} T_j + \omega T_z, \tag{41}$$

in the case of $\Omega \ll f, (D-f)$ and weak coupling. The axial microwave induces another resonance channel at

$$\omega = f + \frac{\Omega_x^2}{2f}, \tag{42}$$

which will merge with $\omega = f + \frac{\Omega_x^2}{4f}$ if the linewidth is larger than $\frac{\Omega_x^2}{4f}$. Now the differential signal will be

$$S(f,t) \approx \frac{1}{16} \left[\left(\frac{\Omega_x d_{zx}}{f} + \frac{\Omega_z d_{xx}}{D-f} \right)^2 + \left(\frac{\Omega_x d_{zy}}{f} + \frac{\Omega_z d_{xy}}{D-f} \right)^2 + \frac{\Omega_z^2 d_{yx}^2}{(D-f)^2} + \frac{\Omega_z^2 d_{yy}^2}{(D-f)^2} \right] \frac{\Gamma_2 t}{{\Gamma_2}^2 + (f-\omega)^2} e^{-\frac{t}{T_{1,\rm NV}}}. \tag{43}$$

For the four peaks in the main text $\omega_1, \omega_2, \omega_9, \omega_{10}$, the relative peak height is $\xi_1 : \xi_2 = 1 : 1.4 \sim 2.2$, and $\xi_9 : \xi_{10} = 1 : 1.5 \sim 3.1$, where the range depends on Ω_x/Ω_z. We note the effect of axial microwave is negligible for low-frequency signals, such as P1 centers or nitroxide radicals. If using the high-frequency detuned part $B_1 \cos{(D+f)t}$, the axial microwave is also negligible.

Supplementary Note 6. EPR measurements on other NDs

To confirm that the ND-EPR spectrum of P1 centers is indeed independent on the orientation of NDs, we repeat the measurement on multiple NDs with random orientations. We use commercially available NDs (MSY-0-0.1, Microdiamant) with an average size of 50 nm. These NDs are first annealed under vacuum at 5×10^{-5} mbar, 800 °C for 2 h to produce stable NV centers. The annealed nanodiamonds are oxidized in air at 550 °C for 2 h to remove the graphitic carbon on the surface, and then acid cleaned. As shown in Supplementary Fig. 6, all these spectra have nearly the same peak positions, although the signal contrasts are different. Moreover, the peak positions are consistent with the theoretical energy splittings of the P1 centers.

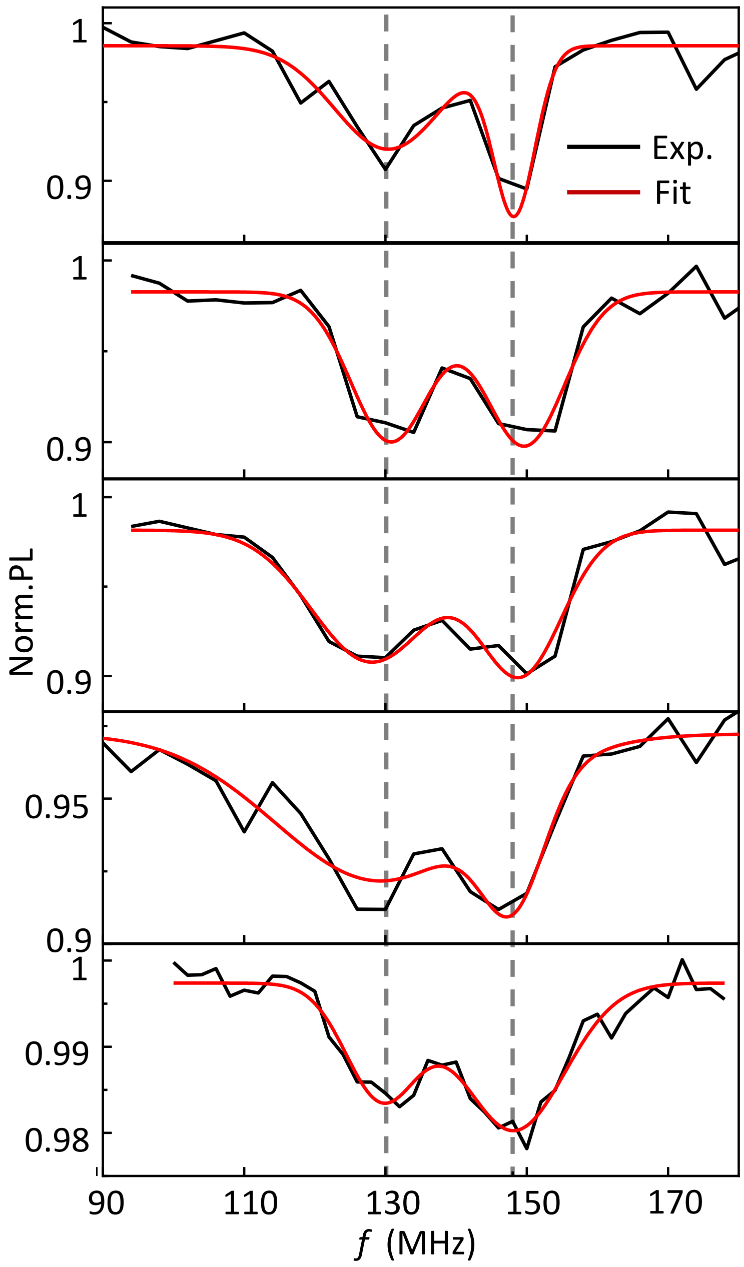

Supplementary Figure 6: **Additional ND-EPR spectra of P1 centers.** The bottom spectra is identical to Fig. 2c in the main text, while the others are measured on a reproduced ND sample. The two vertical dashed lines at 130 MHz and 148 MHz mark the theoretical peak positions of the P1 centers.

The zero-field EPR spectrum of vanadyl ions (Fig. 3d in the main text) is reproducible on multiple NDs, as shown in Supplementary Fig. 7a. All the measurements in this section are performed by using the off-resonance method rather than the amplitude-modulated method. These spectra show similar figure as Fig. 3d in the main text, but with slightly different peak position. We attribute these differences to the different local surface environment of NDs, which leads to different hyperfine constants of vanadyl ions. Since the measurement on signal NDs is time-consuming (nearly one week), all those NDs die before we perform the blank control measurement. Here the dead ND means the hosted NV centers begin losing contrast.

In order to improve the measurement efficiency, we perform the control measurement on ND ensembles. We prepare the sample by: 1. centrifugate 200 μL ND suspension (1 mg/mL) with speed of 16,000 rpm and time of 15 min; 2. remove 190 μL supernatant; 3. take 1 μL condensed ND suspension on the coplanar waveguide; 4. add 2 μL vanadyl sulfate solution on the ND suspension when it is nearly dry; 5. seal the sample to prevent oxidation. For blank control, the vanadyl sulfate in step 4 is removed. The experimental setup is similar with the main text except the photodiode is replaced by one with higher dynamical range (Thorlabs APD410A). The spectrum shows more obvious line broadening, which is reasonable because of the dependence of peak position on NDs. Nevertheless, the control measurement confirms the peaks at 700-1100 MHz indeed come from the vanadyl ions (Supplementary Fig. 7b).

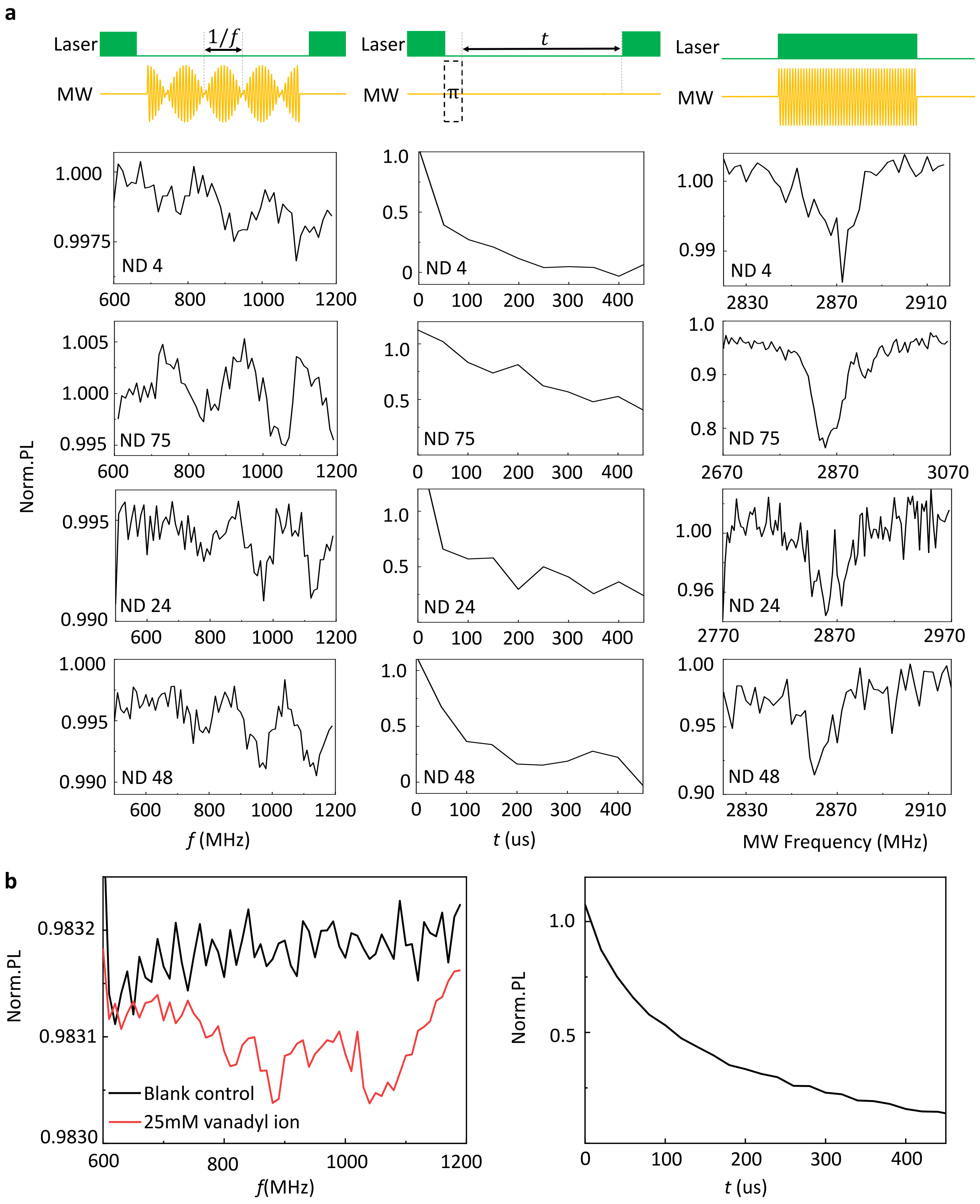

Supplementary Figure 7: **Additional ND-EPR spectra of vanadyl ions.** **a** Measurements on single NDs. Left, middle, and right panels are the EPR spectra of vanadyl ions, T_1 measurements of NV centers, and ODMR spectra of NV centers, respectively. The corresponding pulse sequences are shown on the top. **b** Measurements on ND ensembles. The left is the EPR spectra of vanadyl ions, while the right is T_1 measurements of NV centers.

Supplementary Note 7. Ensemble EPR measurements of vanadyl ions

The vanadyl ions in our measurement exist in glycerol aqueous solution rather than pure water, so the ions may be coordinated by glycerol ligands, resulting in a variation of the hyperfine constants. To see if it happens, we measure the ensemble EPR spectra of vanadyl ions in different solutions. It is difficult to make a direct comparison between the 9:1 glycerol aqueous solution and the pure water, because we can neither acquire liquid EPR spectra in such a viscous solution, nor acquire powder EPR spectra in pure water. To address this issue, we first measure the liquid EPR spectra of 25 mM vanadyl ions in pure water and in 1:3 glycerol aqueous solution on JEOL JES-FA200 ESR spectrometer. As shown in Supplementary Fig. 8a, the two spectra are almost the same, which means a low concentration (25%) of glycerol will not change the coordinate ligands of the vanadyl ions or at least not change the hyperfine constants. We then measure the low-temperature powder EPR spectra of 25 mM vanadyl ions in 1:3 and 9:1 glycerol aqueous solutions on Chinainstru & Quantumtech (Hefei) EPR200-Plus with continues-wave band frequency. As shown in Supplementary Fig. 8b, the two spectra are also nearly the same regardless of the different signal strengths, which means changing the glycerol concentration from 25% to 90% will also not change the hyperfine constants. Therefore, we think that the vanadyl ions have the same hyperfine constants in 9:1 glycerol aqueous solutions as in pure water.

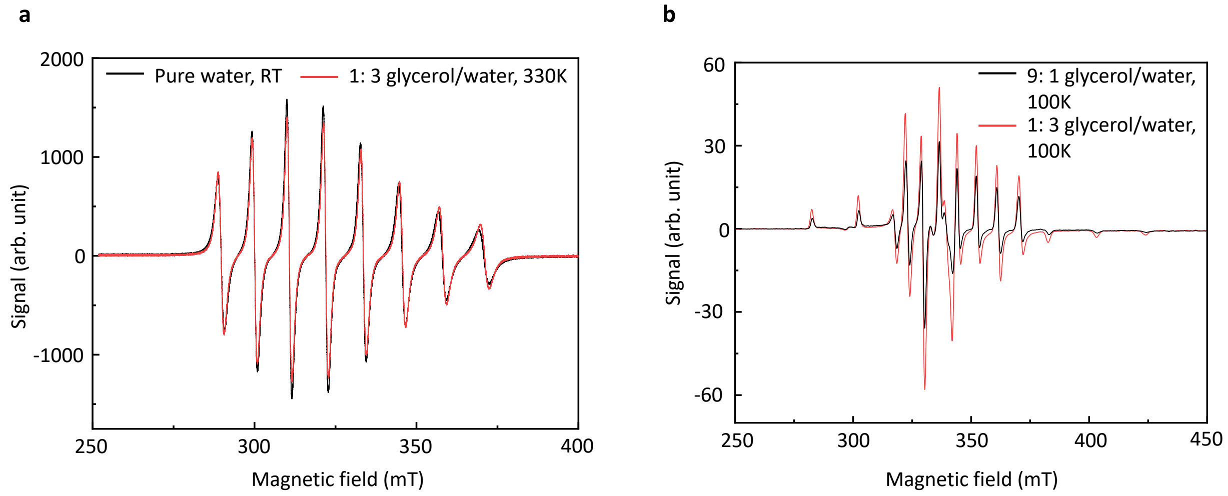

Supplementary Figure 8: **Ensemble EPR spectra of vanadyl ion in different solutions.** **a** Liquid EPR spectra of 25 mM vanadyl ions in pure water and in 1:3 glycerol aqueous solution. The latter is heated to 330 K to have the same viscosity with the pure water. **b** Low-temperature powder EPR spectra of 25 mM vanadyl ions in 1:3 and 9:1 glycerol aqueous solutions.
